# Contribution of Exogenous Genetic Elements to the Group A *Streptococcus* Metagenome

**DOI:** 10.1371/journal.pone.0000800

**Published:** 2007-08-29

**Authors:** Stephen B. Beres, James M. Musser

**Affiliations:** Center for Molecular and Translational Human Infectious Diseases Research, The Methodist Hospital Research Institute, Houston, Texas, United States of America; Centre for DNA Fingerprinting and Diagnostics, India

## Abstract

Variation in gene content among strains of a bacterial species contributes to biomedically relevant differences in phenotypes such as virulence and antimicrobial resistance. Group A *Streptococcus* (GAS) causes a diverse array of human infections and sequelae, and exhibits a complex pathogenic behavior. To enhance our understanding of genotype-phenotype relationships in this important pathogen, we determined the complete genome sequences of four GAS strains expressing M protein serotypes (M2, M4, and 2 M12) that commonly cause noninvasive and invasive infections. These sequences were compared with eight previously determined GAS genomes and regions of variably present gene content were assessed. Consistent with the previously determined genomes, each of the new genomes is ∼1.9 Mb in size, with ∼10% of the gene content of each encoded on variably present exogenous genetic elements. Like the other GAS genomes, these four genomes are polylysogenic and prophage encode the majority of the variably present gene content of each. In contrast to most of the previously determined genomes, multiple exogenous integrated conjugative elements (ICEs) with characteristics of conjugative transposons and plasmids are present in these new genomes. Cumulatively, 242 new GAS metagenome genes were identified that were not present in the previously sequenced genomes. Importantly, ICEs accounted for 41% of the new GAS metagenome gene content identified in these four genomes. Two large ICEs, designated 2096-RD.2 (63 kb) and 10750-RD.2 (49 kb), have multiple genes encoding resistance to antimicrobial agents, including tetracycline and erythromycin, respectively. Also resident on these ICEs are three genes encoding inferred extracellular proteins of unknown function, including a predicted cell surface protein that is only present in the genome of the serotype M12 strain cultured from a patient with acute poststreptococcal glomerulonephritis. The data provide new information about the GAS metagenome and will assist studies of pathogenesis, antimicrobial resistance, and population genomics.

## Introduction

Study of intraspecies variation in chromosomal gene content and sequence diversity has become an area of considerable interest in recent years [1]–[9]. Several factors have prompted research on this topic. First, the genome sequences that are now available for at least one member of many eukaryotic and prokaryotic species provide reference templates for indexing intraspecies diversity (cmr.tigr.org and www.genomesonline.org). Second, analysis of intraspecies genetic diversity is a crucial component of studies designed to understand the molecular basis of phenotypic variation in traits such as organism behavior, disease susceptibility, and response to pharmacologic agents. Third, sequences that are polymorphic within members of the same species are used in molecular epidemiology studies to distinguish among closely related organisms for public health and forensic purposes. Fourth, given that mutation, selection, and inheritance are the basis of evolution, comparison of intraspecies genetic variation provides insights into the molecular processes underlying evolution. A major factor that has contributed to increased interest in species-level genetic diversity among pathogenic microbes is the need to understand the molecular basis of biomedically relevant topics such as strain emergence, virulence differences, disease manifestation, and evolution of pathogenic traits.

The human bacterial pathogen group A *Streptococcus* (GAS) is an ideal model organism for studying molecular processes generating intraspecies genomic diversity and the contribution of specific genetic differences to host-pathogen interactions. GAS causes a wide range of infections, including pharyngitis, cellulitis, sepsis, necrotizing fasciitis, and post-infection sequelae including acute rheumatic fever (ARF) and acute poststreptococcal glomerulonephritis (APSGN) [10]–[13]. For many decades, GAS strains have been classified based on serologic differences in M protein, a highly polymorphic cell-surface virulence factor [14], [15]. More than 125 M protein types and *emm* gene types have been identified (for convenience we will use the terms M protein serotype and *emm* type interchangeably), and the number of subtypes is far higher ([16]; www.cdc.gov/ncidod/biotech/strep/emmtypes.htm). Importantly, epidemiologic studies conducted over many decades have repeatedly found that certain M protein types are non-randomly associated with particular human infections [10], [17]–[22]. For example, serotype M1 and M3 GAS strains commonly cause pharyngitis and invasive infections, and M28 GAS strains are significantly overrepresented among puerperal sepsis and neonatal GAS infections [17], [18], [21]–[25]. Thus, there is a rich phenotypic and clinical framework available for interpreting genome sequence information. Moreover the relatively modest size, low G+C content, single chromosome, rare occurrence of extra-chromosomal elements, and lack of extensive repetitive sequences permits complete GAS genome sequences to be determined at reduced cost, time, and effort relative to many other microbial pathogens [26]–[31]. In addition, the increasing number of reports from many countries describing the emergence of GAS strains resistant to antimicrobial agents such as erythromycin and tetracycline [32]–[44] provides a public health impetus for sequencing the genome of additional strains. In this manuscript we describe new findings based on the genome sequence of serotype M2, M4, and two M12 strains, with a focus on unique gene content encoded by integrated conjugative elements (ICEs).

## Results

### Strain selection

The strains selected for sequencing were chosen on the basis of a variety of characteristics. The primary criterion was that the strains were of an unsequenced M protein serotype that abundantly cause noninvasive and invasive infections. Consistent with this serotype M2, M4 and M12 strains are all common isolates of noninvasive and invasive infections in the United States and other developed countries (see www.cdc.gov/ncidod/dbmd/abcs/survreports.htm). Strains representing these serotypes have not been previously sequenced.

An additional criterion was an epidemiologic association with a distinct pathogenic character in order to facilitate assessment of gene content influencing strain genotype-patient disease phenotype relationships or epidemic behavior. Serotype M2 strain MGAS10270 was obtained from a patient in Texas with pharyngitis in the late 1990s. Serotype M2 strains are associated with female uritogenital tract infections [18], [45], [46]. Serotype M4 strain MGAS10750 was cultured from a patient with pharyngitis in Florida in 2001. This strain is resistant to erythromycin (MIC 1 µg/ml) and is PCR positive for the *erm*(A) gene. Erythromycin resistant serotype M4 strains have caused epidemic outbreaks of infection [18], [47], [48]. Serotype M12 strain MGAS2096 was isolated from a patient with poststreptococcal glomerulonephritis in Trinidad in 1960. The isolation of GAS from patients with APSGN is rare as in most cases the infection has cleared prior to glomerulonephritis manifestation. This organism, also known as strain A374, has been studied previously [49]–[51]. Given that serotype M12 strain MGAS2096 was isolated over 45 years ago, and changes in prophage content have been associated with rapid shifts in GAS pathogenesis, we also elected to sequence a contemporary M12 strain for comparison. Serotype M12 strain MGAS9429 was cultured from a pediatric patient with pharyngitis in the Texas in 2001. Strain MGAS9429 has the most common prophage virulence gene profile detected from among 33 contemporary serotype M12 strains studied (J.M.M., unpublished data).

A repeated epidemiological finding dating back to the 1930s is that strains of certain M protein serotypes are nonrandomly associated with the poststreptococcal infection sequela, ARF and APSGN [11], [12], [52], [53]. Observations of distinct differences in disease manifestation from these studies lead to the supposition that rheumatogenicity and nephritogenicity may be independent properties of two separate GAS genetic lineages that broadly correspond with strains most commonly causing throat and skin infections, respectively. The presence or absence of the serum opacity factor (*sof*) gene encoding for a lipoproteinase that confers the ability to opacify human serum is considered a marker of the two lineages [54], [55]. Excepting serotype M28 strain MGAS6180, all of the previously sequenced GAS strains are *sof* negative and represent serotypes considered rheumatogenic (2 each of M1 and M3, and one each of M5, M6, and M18) (Table 1). The four strains selected for sequencing and described here are all *sof* positive representing serotypes considered nephritogenic. Thus in addition to their other disease associations, these four genomes also provide data for assessing genetic differences between the posited GAS skin/nephritogenic and throat/rheumatogenic lineages.

### Overview of general genome features

**Figure 1:**
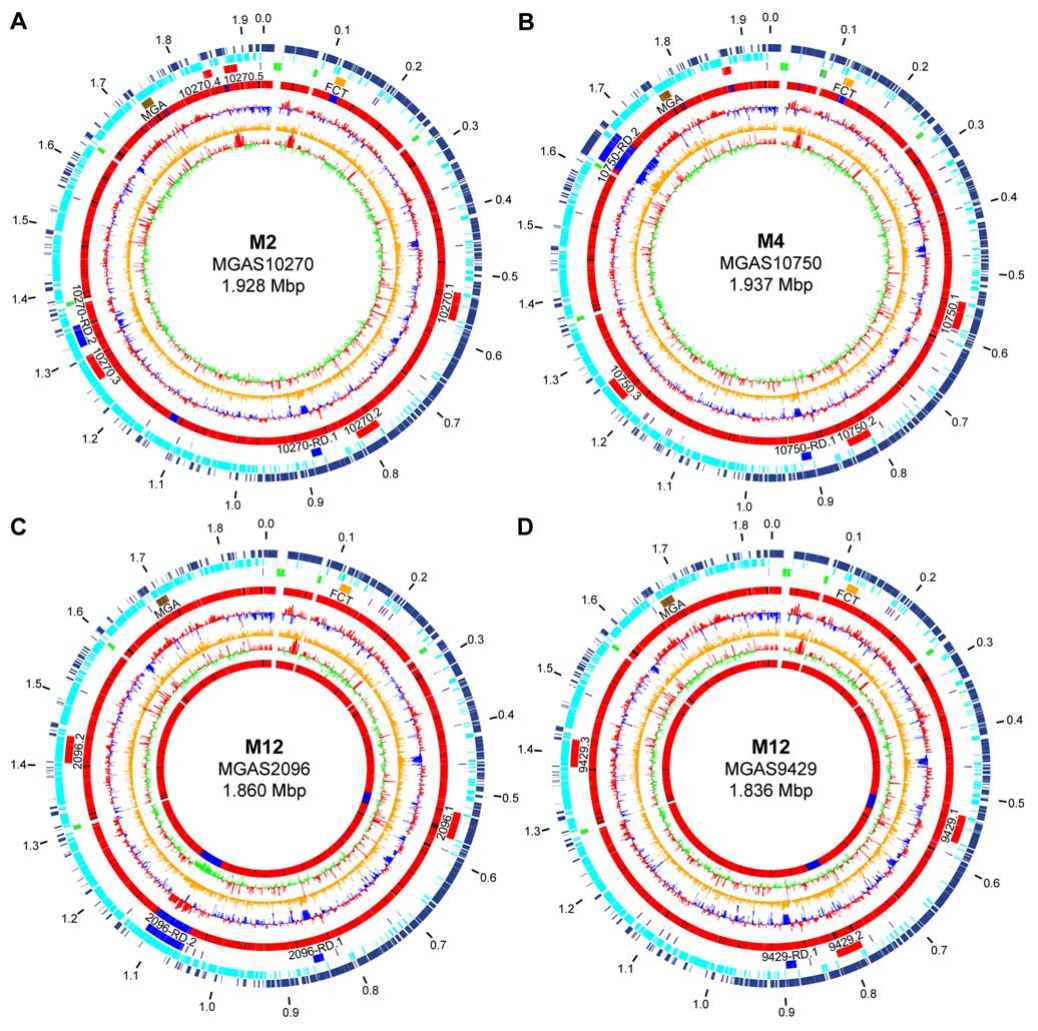
Genome circular atlases. (A) MGAS10270, (B) MGAS10750, (C) MGAS2096, and (D) MGAS9429. Data from outermost to innermost circles are in the following order. Genome size in mega base pairs (circle 1). Annotated coding sequences on the forward (circle 2) and reverse strands (circle 3) are in dark and light blue, respectively. Reference landmarks (circle 4) illustrated are: ribosomal RNAs in green, FCT region in gold, transposons in purple, prophages in red, ICEs in royal blue, and Mga regulon region in brown. Comparison of gene content to the 11 other sequenced GAS strains (circle 5) is given as a gradient of nucleotide sequence similarity from low in blue to high in red. CDS percent G+C content (circle 6) with greater than and less than average in red and blue, respectively. Net divergence of CDS dinucleotide composition (circle 7) from the average is in orange. Codon adaptation index, that is codon use consistent with that of highly expressed genes (circle 8) with greater than and less than average in red and green, respectively. Additionally for the two serotype M12 strains a comparison of gene content relative to each other (circle 9) is given as a gradient of nucleotide sequence similarity from low in blue to high in red.

Consistent with the genomes of eight previously sequenced GAS strains [26]–[31], [56], the serotype M2, M4 and both M12 genomes each is a single circular chromosome of ∼1.9 Mb (Table 1, Fig. 1). The percent G+C content of these genomes is approximately 38.5%, essentially identical to the other eight sequenced GAS genomes (range, 38.31%–38.73%). Each of these genomes has six operons encoding adjacent 5S, 16S, and 23S ribosomal RNAs. Each has the multi-locus sequence type (MLST) that is most common for their M type [57]. Predicted coding sequence composes a similar portion of each of the genomes. Among the 12 sequenced GAS genomes, on average coding sequence constitutes 86.4% of each genome, with a mean gene size of 870 nt.

**Table 1:** Characteristics of Sequenced GAS Strains

Strain	M type	MLST	*sof*	Size, bp	% G+C	CDS		Elements	
						No.	%	Phage	ICE
SF370	1	28	neg	1,852,441	38.51	1,697	83.79	4	1
MGAS5005	1	28	neg	1,838,554	38.53	1,865	86.39	3	1
MGAS10270	2	55	pos	1,928,252	38.43	1,987	87.18	5	2
MGAS315	3	15	neg	1,900,521	38.59	1,865	85.76	6	0
SSI-1	3	15	neg	1,894,275	38.55	1,861	84.61	6	0
MGAS10750	4	39	pos	1,937,111	38.31	1,979	87.16	4	2
Manfredo	5	99	neg	1,841,271	38.62	1,803	86.72	5	0
MGAS10394	6	382	neg	1,899,877	38.69	1,886	87.28	8*	0*
MGAS2096	12	36	pos†	1,860,355	38.73	1,898	87.39	2	2
MGAS9429	12	36	pos†	1,836,467	38.54	1,878	87.81	3	1
MGAS8232	18	42	neg	1,895,017	38.55	1,845	85.23	5	0
MGAS6180	28	52	pos	1,897,573	38.35	1,894	87.09	4	3

* Element 10394.4 is composite element that has a phage-like 5′ end and an ICE-like 3′end.

† Strains MGAS2096 and MGAS9429, like most serotype M12 strains, although *sof* positive by PCR are phenotypically Sof negative due to internal gene mutations.

The majority of each genome (>85%) is conserved in gene content and context relative to the others (Fig. 1). This core sequence is conserved at greater than 98% nucleotide identity and comprises the endogenous “core” of the GAS metagenome (i.e., the common part of the chromosome that does not include obvious exogenous genetic elements such as prophages and ICEs). The endogenous core encodes many proven or putative secreted virulence factors, including M protein, streptolysin O, streptolysin S, streptokinase, pyrogenic toxin superantigens (SmeZ), collagen-like proteins (SclA and SclB), and proteases (SpeB, Mac, and ScpA) [11], [12] to name a few. The average size of the 12 sequenced genomes is 1,882 kb, and the difference between the smallest and largest genome is 100.6 kb or 5.3% of the average size. The extent of size variation in the GAS genomes is similar to that reported for *Staphylococcus aureus* genomes, greater than found in *Chlamydia trachomatis* and *Mycobacterium tuberculosis*, and considerably less than for certain *Escherichia coli* strains [1], [2], [4]–[7], [9], [58].

### Overview of exogenous genetic elements, prophages, and ICEs

**Figure 2:**
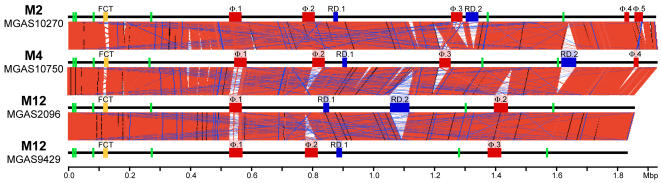
Aligned GAS genomes. Illustrated are linear diagrams of the four newly determined GAS genome sequences and regions of conserved gene content in pair-wise comparisons. Shown for each genome diagram in green are the six rRNA operons, in red are prophages, and in blue are ICEs. Whole-genome comparisons were made using BLASTN (www.webact.org, e = 1×10^−4^, word size = 18 nt) and the graphic depictions of the alignments were made using the Artemis Comparison Tool (www.sanger.ac.uk/Software/ACT/). Regions of conserved syntenic gene content are indicated by blocks of salmon linking the stacked genome diagrams. Nearly all regions of discontinuity in the genome alignments are attributable to exogenous genetic elements.

**Figure 3:**
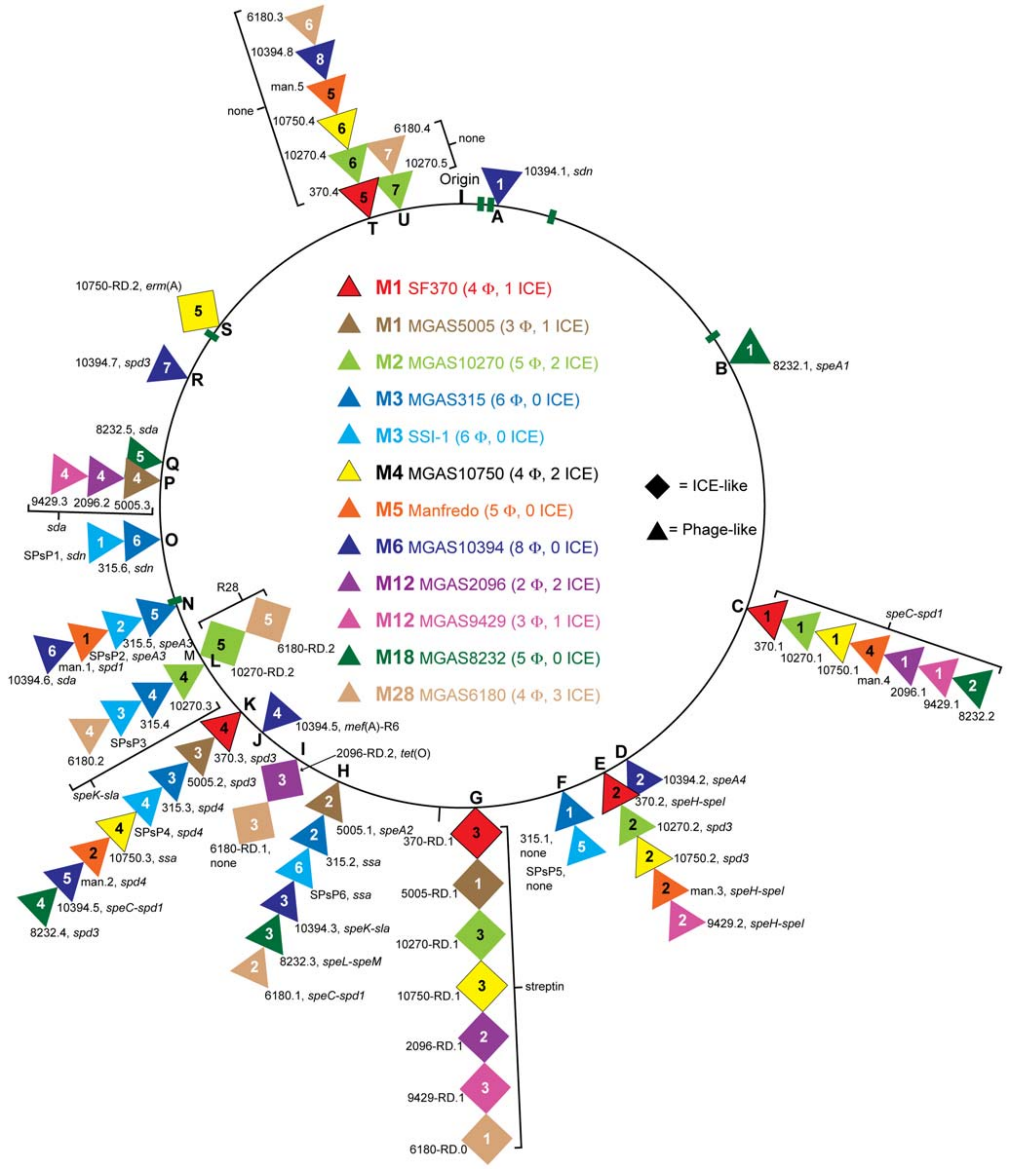
GAS metagenome exogenous elements. Illustrated are loci of integration of phages and ICEs into the core chromosome. Prophages are indicated with triangles and ICEs with squares. Stacked triangles and squares indicate a common integration site. Elements are color-coded to indicate the source strain. Prophages and ICEs are numbered as they occur clockwise around the core chromosome for each strain. Integration loci are lettered alphabetically as they occur clockwise around the core chromosome. The six rRNA operons are shown as green bars. Gene designations are as follows: 1) secreted pyrogenic-toxin-superantigens: *speA*, *speC*, *speH*, *speI*, *speK*, *speL*, *speM*, and *ssa*; 2) secreted DNAses: *sda*, *sdn*, *spd1*, *spd3*, and *spd4*; 3) secreted phospholipase: *sla*; 4) antimicrobial resistance: *erm*(A), *mef*(A), and *tet*(O); 5) cell surface adhesins: R6 and R28; 6) none, these elements lack a known or obvious virulence gene.

To identify regions of difference among the sequenced GAS genomes they were aligned pair-wise. This revealed regions (5 kb–63 kb) differing in gene content and/or context that disrupted the continuity of the aligned sequences (this is illustrated for the four newly sequenced genomes in Fig. 2). Bioinformatic analysis found that these regions of difference contain gene content similar to prophages and ICEs. Twenty-one exogenous genetic elements (14 prophage-like and 7 ICE-like) ranging from 12 kb to 63 kb in size were identified in the serotype M2 (5 Φ, 2 ICE), M4 (4 Φ, 2 ICE), and M12 strains MGAS2096 (2 Φ, 2 ICE) and MGAS9429 (3 Φ, 1 ICE) genomes (Fig. 1, Table 2). In total, we identified 67 obvious exogenous genetic elements (55 prophages and 12 ICEs) integrated at 21 distinct loci of the core chromosome in the 12 GAS genomes (Fig. 3, Table 2). Based on gene content, some of the smaller elements likely are remnants of ancestral genetic elements that have undergone reductive evolution. However, we cannot exclude the possibility that these elements are mobile and were acquired by lateral gene transfer. As most of these exogenous genetic elements have not been shown experimentally to be transferable we refer to them as putatively-mobile.

**Table 2:** Characteristics of GAS Exogenous Elements

M-type, Strain		Exogenous Element	Virulence Gene(s)	CDS Start	CDS Stop	Size (kbp)	Percent G+C
**M1, SF370**							
		370.1	*speC-spd1*	0655	0712	40.9	39.3
		370.2	*speH-speI*	0937	1008	42.5	38.0
		370-RD.1	*srtA*	1075	1088	12.2	33.2
		370.3	*spd3*	1436	1488	33.5	38.0
		370.4	none*	2122	2147	13.6	36.8
**M1, MGAS5005**							
		5005-RD.1	*srtA*	0797	0816	12.2	33.2
		5005.1	*speA2*	0995	1052	38.1	38.0
		5005.2	*spd3*	1168	1222	33.4	37.7
		5005.3	*sda*	1414	1467	40.8	39.8
**M2, MGAS10270**							
		10270.1	*speC-spd1*	0536	0598	44.1	38.0
		10270.2	*spd3*	0796	0853	37.9	38.2
		10270-RD.1	*srtA*	0910	0932	17.5	31.9
		10270.3	*speK-sla*	1297	1361	41.8	38.5
		10270-RD.2	R28	1378	1411	34.7	34.5
		10270.4	none	1874	1896	14.3	36.9
		10270.5	none	1917	1951	11.8	36.6
**M3, MGAS315**							
		315.1	none	0681	0736	39.5	37.8
		315.2	*ssa*	0919	0978	41.1	38.3
		315.3	*spd4*	1094	1145	34.4	38.0
		315.4	*speK-sla*	1203	1266	61.8	38.6
		315.5	*speA3*	1300	1354	38.2	38.0
		315.6	*sdn*	1408	1458	40.0	39.7
**M3, SSI-1**							
		SPsP1	*sdn*	0408	0456	40.0	39.7
		SPsP2	*speA3*	0507	0561	38.2	38.1
		SPsP3	*speK-sla*	0597	0659	41.8	38.6
		SpsP4	*spd4*	0717	0771	34.4	38.0
		SPsP5	none	0877	0937	41.0	38.2
		SPsP6	*ssa*	1118	1172	39.9	37.7
**M4, MGAS10750**							
	10750.1		*speC-spd1*	0560	0622	43.8	38.0
	10750.2		*spd3*	0831	0889	37.9	38.3
	10750-RD.1		*srtA*	0945	0967	17.6	31.9
	10750.3		*ssa*	1276	1328	35.6	38.0
	10750-RD.2		*erm*(A)	1679	1719	48.9	31.2
	10750.4		none	1897	1921	13.9	35.2
**M5, Manfredo**							
	man.1		*spd3*	0471	0535	41.4	38.6
	man.2		*spd4*	0631	0692	36.0	37.9
	man.3		*speH-speI*	1021	1070	39.9	38.2
	man.4		*speC-spd1*	1263	1322	40.7	38.2
	man.5		none	1764	1779	12.3	36.9
**M6, MGAS10394**							
	10394.1		*sdn*	0020	0068	39.9	39.7
	10394.2		*speA4*	0733	0741	8.8	32.2
	10394. 3		*speK-sla*	0982	1026	33.2	38.0
	10394.4†		*mef*(A), R6	1123	1173	58.9	38.2
	10394.5		*speC-spd1*	1194	1242	31.9	38.4
	10394.6		*sda*	1338	1366	24.5	40.4
	10394.7		*spd3*	1540	1562	21.1	39.5
	10394.8		none	1804	1824	13.9	36.6
**M12, MGAS2096**							
	2096.1		*speC-spd1*	0553	0602	41.3	39.1
	2096-RD.1		*srtA*	0869	0890	17.5	31.9
	2096-RD.2		*tet*(O)	1103	1159	63.0	43.1
	2096.2		*sda*	1440	1492	40.8	39.8
**M12, MGAS9429**							
	9429.1		*speC-spd1*	0532	0594	41.3	38.0
	9429.2		*speH-speI*	0795	0851	17.5	38.1
	9429-RD.1		*srtA*	0911	0934	63.0	31.9
	9429.3		*sda*	1415	1468	40.8	39.8
**M18, MGAS8232**							
	8232.1		*speA1*	0336	0394	39.0	37.7
	8232.2		*speC-spd1*	0716	0779	41.1	38.2
	8232.3		*speL-speM*	1238	1309	46.7	37.6
	8232.4		*spd3*	1444	1506	38.4	37.7
	8232.5		*sda*	1745	1808	41.8	38.5
**M28, MGAS6180**							
	6180-RD.0		*srtA*	0771	0793	17.5	31.9
	6180.1		*speC-spd1*	0967	1033	46.3	37.2
	6180-RD.1		none	1079	1089	11.1	30.7
	6180.2		*speK-sla*	1220	1285	42.3	38.4
	6180-RD.2		R28	1302	1337	36.3	35.0
	6180.3		none	1789	1813	14.3	36.8
	6180.4		none	1840	1864	11.8	36.6

* There are no known or obvious virulence genes.

† Element 10394.4 is chimeric, composed of a phage-like 5′ end and an ICE-like 3′end.

All of the sequenced GAS strains have multiple prophages, most of which encode one or two proven or putative secreted virulence factors. Prophages constitute ∼10% of any one genome and are the major contributor to variation in gene content among the sequenced GAS genomes [59]. As such phage have been a major source of virulence factors uniquely present in each of the genomes. In opposition to this prior trend, no new putative secreted virulence factors were encoded by the 14 prophages present in the serotype M2, M4, and M12 genomes sequenced. Importantly however, this does not mean that the prophages in these genomes are identical to those in the other sequenced GAS genomes, or that no new secreted putative virulence genes were identified in these strains. To the contrary, the apparent mobility and highly recombinogenic mosaic structures of the prophages and ICEs in the GAS genomes results in each sequenced GAS genome having a unique complement of exogenous elements and secreted virulence determinants.

In addition to multiple prophages, seven of the sequenced strains also contain large (5 kb–63 kb) regions that have features of ICEs [60], [61]. The presence of ICEs in the newly sequenced strains means that conjugative lateral gene transfer is a second important contributor to GAS metagenome diversification. For example, ICEs, 2096-RD.1 and 2096-RD.2, present in the genome of serotype M12 strain MGAS2096 account for half (49.5%) of the total of 162.6 kb of foreign sequence identified in this strain. Notably, ICEs are more prevalent among the *sof* positive strains (averaging 2.0 per genome) than the *sof* negative strains (averaging 0.3 per genome) (Table 1). Each of the five *sof* positive genomes (M2, M4, 2 M12 and M28) contain one or more ICEs, accounting for 10 of the 12 ICEs among the sequenced strains. In contrast among the 7 *sof* negative genomes (2 M1, 2 M3, M5, M6, and M18) only the two serotype M1 strains have an ICE. Furthermore, unlike the prophage which on average differ only 0.5 percent in G+C content (ave. = 38.05) from the GAS endogenous core genome (ave. = 38.61), the ICEs differ by an average of 5 percent (ave. = 33.62) (Table 2).

To identify new GAS metagenome gene content, the predicted CDSs of each newly sequenced strain was compared to the genomes of the eight previously sequenced GAS strains using BLASTN. In total 242 genes were identified that shared less than 50% overall nucleotide identity with sequence of any of the previously determined genomes (supplementary Table S1). ICE-like regions accounted for 41% (98/242), the fibronectin-collagen-T antigen encoding (FCT) region 9% (22/242), and prophages 14% (33/242) of this new gene content (supplementary Table S1). Given that ICE-like elements and the FCT region encode half of this newly identified GAS metagenome gene content, the description of the M2, M4, and M12 genome sequences that follows will focus on these components. The ICEs will be presented in order as they occur integrated clockwise around the GAS metagenome (Fig. 3). Specific prophages in these genomes will not be described as no new prophage associated putative virulence genes were found in these genomes, and the contribution of phage to GAS pathogenesis and genome diversification has been the subject of recent reviews [59], [62]–[64].

### Streptin production ICE-like region

The genome of the serotype M2, M4, and M12 strains each has an ∼15 kb ICE-like region of difference (designated 10270-RD.1, 10750-RD.1, 2096-RD.1, and 9429-RD.1) composed of 9 or 10 genes (srt genes) encoding proteins mediating production of streptin, a lantibiotic bacteriocin (for a diagram of the srt gene locus see [65]) (Fig. 3 site G). These genes are flanked on the 5′ and 3′ sides by multiple CDSs with similarity to ICE relaxases and site-specific recombinases, respectively. This element is integrated between rpiL and dacA1 (SF370: Spy1073&Spy1093), genes encoding a ribosomal large subunit protein and peptidoglycan synthesis transpeptidase, respectively. The integration of this element appears to result in deletion of a ∼200 nt region located between these two genes. Genes encoding for streptin production are also present at this loci in the two serotype M1 and the M28 genome sequences. However due to multiple internal deletions the streptin ICE-like region present in the genome of the two serotype M1 strains is only ∼10 kb in size. The genome sequences of the serotype M3, M5, M6, and M18 strains (all sof negative) lack an analogous streptin ICE-like region. The G+C content of this element in all 5 strains with the ∼15 kb intact form is 31.9%, a value considerably less that the 38.5% GAS genome average, consistent with interspecies gene horizontal transfer.

Analysis of bacteriocin production by GAS strains of many different serotypes has found that serotype M2, M12, and M28 strains produce a bacteriocin resulting in a P-type 777 growth inhibition profile on indicator strains, whereas serotype M1, M3, M4, M5, M6, and M18 strains do not [65]. Thus, with the exception of serotype M4 strains, the results parallel the distribution of the 15-kb form of this ICE among the sequenced GAS genomes. Serotype M4 strains have a unique bacteriocin growth inhibition profile, P-type 655 attributed to the production of both streptin and salivaricin A [65], [66]. Consistent with this finding, the M4 strain MGAS10750 genome has an intact ∼10 kb locus of seven genes (salR-K-Y-X-T-M-A; MGAS10750_Spy1722-28), encoding for production of salivaricin (for a diagram of the sal gene locus see ref. 116). The salivaricin genes are located 3′ adjacent to 10750-RD.2 (Fig. 3 site S). A homologous gene locus is present in the other sequenced GAS strains but all have deletions in salT and/or salM that preclude SalA production [66]. Although the 30.5% G+C content of this region suggests it is not endogenous to the GAS genome, it lacks gene content characteristic of either phage or ICE.

The streptin element in the 5 strains in which it is intact has an average of 1 SNP every 175 nt. This level of sequence polymorphism is similar to the average of 1 SNP every ∼120 nt frequency present serotype-to-serotype among the sequenced GAS strain core chromosomes. In addition, very few SNPs present in this element were common to strains of different M protein serotypes. These genetic features strongly favor the likelihood that these sof positive serotypes, each possessing the streptin gene locus, share a common ancestor. That is, acquisition of the streptin ICE by lateral transfer occurred before the divergence of the genomes of distinct M protein serotypes that contain this element.

### Exogenous genetic element 2096-RD.2 encoding tetracycline resistance

Although most studies of antimicrobial agent resistance in GAS have focused on macrolides such as erythromycin, increasing emphasis is being placed on analysis of strains resistant to tetracycline, either alone or in combination with macrolides [67]. Tetracycline resistance in GAS is mediated either by *tet*(M) or *tet*(O), genes which encode proteins that protect the ribosome [67]. The occurrence of the *tet*(M) gene in GAS has been known for some time, whereas the presence of the *tet*(O) gene in this species was reported relatively recently [68], [69].

**Figure 4:**
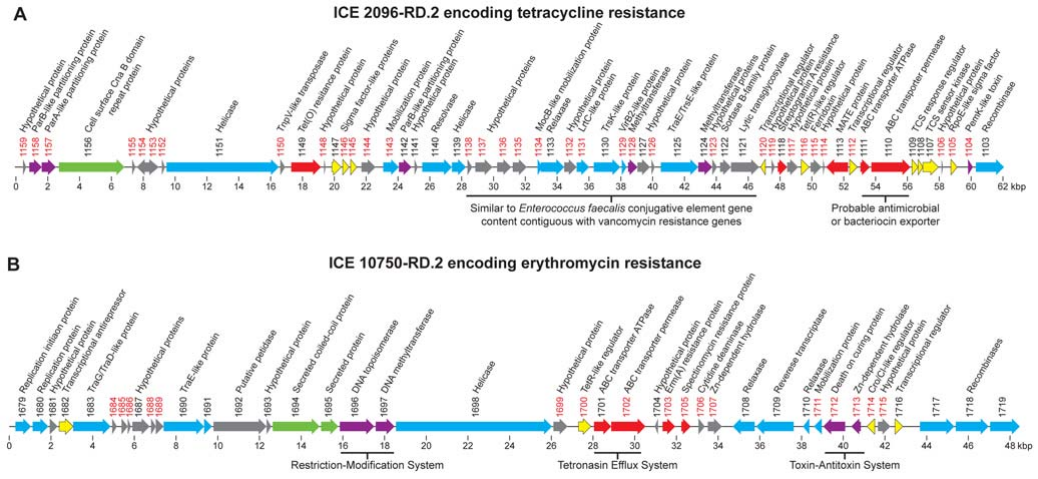
ICEs encoding antimicrobial resistance genes. (A) 2096-RD.2 encoding Tet(O). (B) 10750-RD.2 encoding Erm(A). Illustrated are predicted coding sequences with gene numbers and predicted functions. Gene numbers given in red denote unique gene content as determined by BLASTP comparison to the GAS metagenome (no hit at e = 1×10^−6^). CDS are color coded to designate functionally related groups: red, antimicrobial resistance; green, secreted and cell surface; blue, mobilization and transfer; violet, element maintenance; yellow, transcriptional regulation; grey, hypothetical and unclassified.

Our primary impetus for sequencing the genome of serotype M12 strain MGAS2096 was its documented association with acute poststreptococcal glomerulonephritis. We found that this genome has a unique 63-kb ICE-like element (designated 2096-RD.2) encoding several antibiotic resistance genes including *tet*(O) (Fig. 4 panel A). This ICE-like element is both the largest and has the highest G+C content (43%) of the exogenous elements present in the 12 sequenced GAS strains (Table 2). 2096-RD.2 is integrated at the 3′ end of a tRNA uracil methyltransferase gene (SF370: Spy1346). 6180-RD.1 occupies the analogous site in the genome of the sequenced serotype M28 strain, however these two ICEs have almost no genes in common (Table 2, Fig 3). The *tet*(O) gene (MGAS2096_Spy1149) encoded by 2096-RD.2 is >98% identical to *tet*(O) gene found in *Streptococcus mutans*, *Streptococcus pneumoniae*, and *Campylobacter jejuni*. 2096-RD.2 also has an acetyltransferase gene (MGAS2096_Spy1118) encoding a product with 47% identity and 66% similarity to Vat(B), a protein conferring resistance to streptogramin A in *Staphylococcus aureus* [70]. In addition, the 2096-RD.2 element has a gene (MGAS2096_Spy1113) that encodes a hydrophobic protein with ∼65% amino acid similarity to Na^+^-driven multi-drug efflux pumps (MATE proteins) found in *Clostridium tetani, Listeria monocytogenes,* and *Porphyrmonas gingivalis,* and several other species of pathogenic bacteria. Thus 2096-RD.2 has multiple genes which likely confer resistance to antimicrobial agents.

**Figure 5:**
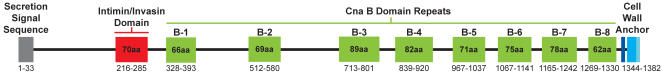
Domain architecture of putative cell surface acidic protein MGAS2096_Spy1156. The protein has a conventional Gram-positive secretion signal sequence and a tripartite (TPKTG, membrane span, positively charged anchor) cell wall attachment domain. The aminoterminal portion of the protein has an intimin/invasin-like domain (Structural Classification of Proteins superfamily: SSF49373) and shares similarity (45% from amino acid ∼50-to-350) with a putative cell surface protein of unknown function (lmo1115) in the genome sequence of the intracellular pathogen *Listeria monocytogenes* strain EGD-e. The carboxyterminal portion of the protein (∼315-to-1350) has 8 Cna B-type domain repeats (Protein Family: PF05738) and shares similarity with multiple proteins annotated as collagen-binding. These characteristics suggest this protein may function as an adhesin/invasin.

Notably, 2096-RD.2 also has a 4,146-bp gene (MGAS2096_Spy1156) encoding a large predicted exported protein with an aminoterminal secretion signal sequence and a carboxyterminal cell-wall anchoring motif (TPKTG) (Fig. 5). This ∼150 kDa acidic protein has a pI of 4.4, and one-fourth of its 1382 amino acids have charged side-chains. Amino acid residues 328–1330 have similarity to Cna, a collagen-adhesion virulence factor and vaccine candidate made by *S. aureus* [71]. The similarity is due mainly to eight regions of ∼75 amino acids each resembling the B domain of Cna that form a beta sandwich extended stalk structure [72], [73]. The amino-terminal end of the mature protein (residues 33–300), although lacking significant similarity to proteins of known function, has a 70 amino acid invasin domain as defined by the intimin protein of enterohemorrhagic and enteropathogenic *Escherichia coli*. Based on these domain similarities MGAS2096_Spy1156 may function as a cell surface adhesin/invasin. Inasmuch as serotype M12 strain MGAS2096 was cultured from a patient with APSGN, and that the 2096-RD.2 ICE is not present in the other sequenced GAS strains including the M12 pharyngitis isolate MGAS9429, the unique proteins encoded by this element warrant further investigation in the context of glomerulonephritis pathogenesis.

### Genetic element 10270-RD.2 and association with puerperal sepsis

The genome of serotype M2 strain MGAS10270 has a 35-kb ICE-like region of difference (designated 10270-RD.2) that is virtually identical to a recently-described exogenous genetic element (6180-RD.2) present in the genome of all serotype M28 strains [29]. An analogous ICE is not present in the genome of the other 10 sequenced GAS strains (Fig 3 site M). ICE 10270-RD.2 is integrated into a tRNA-Thr, is ∼35% G+C, is flanked by 16-bp direct repeats, and has seven genes encoding proteins with predicted secretion signal sequences. Included among these proteins are cognates of Spy1325 and R28 (MGAS10270: Spy1399 and Spy1410, respectively). Spy1325 and R28 are cell surface anchored adhesins that are expressed during the course of human infection, and are immunoprotective in mouse models of infection [74], [75]. Importantly 10270-RD.2 and 6180-RD.2 are closely related to genetic elements present in strains of group B *Streptococcus*, the leading cause of maternal-neonatal infections in the United States and elsewhere. Including R28, four of the seven inferred extracellular proteins encoded by 6180-RD.2 are made during GAS infection [74], [75]. Excluding differences in the number of repeat domains in the gene encoding R28, 2096-RD.2 and 6180-RD.2 differ by only 8 SNPs. This is one SNP on average every ∼4.4-kb, a frequency 38-fold lower than the core chromosome of strains MGAS2096 and MGAS6180. The very high level of sequence similarity between 2096-RD.2 and 6180-RD.2 means these elements descend from a recent common ancestor and have undergone lateral gene transfer. The occurrence of this ICE in the M2 and M28 clonal lineages is noteworthy because these serotypes have been repeatedly nonrandomly associated with GAS maternal-fetal infections [17], [18], [46], [76]. This epidemiological association and the similarity with sequences of GBS, implicate the 2096-RD.2/6180-RD.2 element in contributing to maternal-fetal infections caused by serotype M2 and M28 strains.

### Element 10750-RD.2 encoding erythromycin resistance

Resistance of GAS to macrolide antibiotics has increased dramatically in the last 10 years and is now a worldwide problem [38]. The great majority of macrolide-resistant GAS strains have either the *mef*(A) gene encoding a drug efflux pump (M resistance phenotype) or the *erm*(A) gene encoding an erythromycin ribosome dimethyltransferase (MLS_B_ resistance phenotype) that modifies a highly conserved adenine residue located in the target bacterial 23S rRNA [77], [78]. Macrolide resistance has been reported to be transferable by conjugal plasmids, phages, and conjugative transposons [68], [79]–[84]. Consistent with these observations, the *mef*(A) and *erm*(A) genes each has been found in association with a very large number of distinct *emm* types [32]–[34], [43], [44], [85]–[87]. In addition, we recently reported that the *mef*(A) gene of macrolide resistant M6 strain MGAS10394 is encoded by an unusual 58.8-kb chimeric genetic element (Fig. 3 site J) with conjugative transposon and prophage characteristics [26], [85].

One of our motivations for sequencing the genome of *erm*(A)-positive serotype M4 strain MGAS10750 was to characterize the genetic element containing the macrolide resistance-conferring gene in this strain. The *erm*(A) gene was present on a 49-kb exogenous element designated 10750-RD.2 that is integrated into the *hsdM* gene encoding host DNA restriction-modification methyltransferase (SF370: Spy1906) (Fig. 3 site S, Fig. 4 panel B). The gene content of this ICE is largely unique to the strain MGAS10750 genome. The *erm*(A) gene in this strain is identical to the *erm*(TR) sequence initially reported in GAS by Seppala et al. [78], and its product is 81.1% identical to Erm(A) of *S. aureus* [88]. Just 5′ of *erm*(A) are two adjacent CDS (SpyM4_1701 and 1702) that encode the ATP-binding and membrane permease components of an ABC transporter. The products of these genes have 66.7% and 44.5% similarity with TnrB2 and TnrB3 respectively, of *Streptomyces longisporoflavus* a producer of tetronasin, a polyether-ionophore antibiotic. TnrB2 and TnrB3 form an ATP-dependent efflux system that confers resistance to tetronasin [89]. Just 3′ of *erm*(A) is a CDS (SpyM4_1705) predicted to encode a phosphotransferase. The product of this gene has 25.6% identity and 39.9% similarity with the last 220 amino acids (residues 122–340) of the spectinomycin resistance aminoglycoside phosphotransferase gene, *aph*, of *Legionella pneumophila* [90]. This conserved region includes the catalytically important residues defined for Aph(3) and Aph(9) [91].

Two of the 10750-RD.2 element CDSs are predicted to encode proteins with conventional gram-positive secretion signal sequences, suggesting that they are secreted extracellularly (Fig. 4 panel B). SpyM4_1694 is predicted to encode a hydrophilic mature protein (residues 31–783) of 87.7 kDa. This protein is of unknown function but has similarity with many proteins including M protein due to the presence of a central (∼250–525 aa) laminin- and myosin-like coiled-coil domain (PFAM: PF00608 and PF01576 respectively). Notably, the top 10 alignments identified using BLASTP to compare SpyM4_1694 with the NCBI non-redundant sequence database are eukaryotic not prokaryotic proteins. SpyM4_1695 is predicted to encode a mature acidic protein (residues 27–289, pI 4.2) of 29.7 kDa. The function of this protein is unknown. SpyM4_1695 lacks significant similarity to proteins of known function, and to known protein domains as determined using either EMBL InterPro- or NCBI conserved domain-searches. Inasmuch as the majority of secreted and cell surface proteins identified in GAS have proven or putative roles in host-pathogen interaction, these predicted extracellular proteins are candidates for further investigation.

### Fibronectin-binding collagen-binding T antigen (FCT) gene region

**Figure 6:**
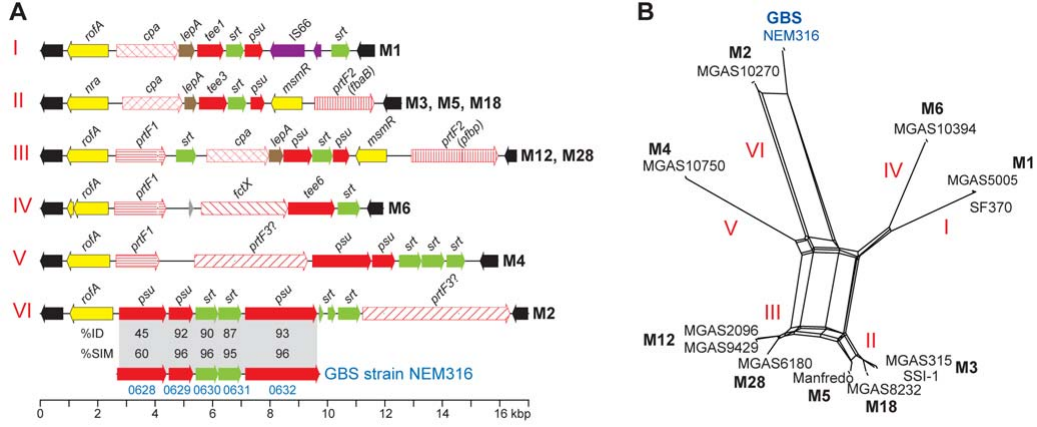
GAS metagenome FCT region variants. (A) Architecture of the FCT region variants. CDSs are colored to designate the following groups: black, conserved flanking genes (SF370: 5′ Spy_0123 and 3′ Spy_0136); yellow, transcriptional regulators; red, extracellular matrix-binding and/or pilin-subunit proteins; tan, signal peptidases; green, sortases; purple, insertion sequences. Although there are differences both intra- and interserotype indicative of antigenic variation, nearly all of the extracellular matrix-binding proteins and pilin-subunit proteins have predicted secretion signal sequences and cell wall attachment domains in one or more of the genomes. Additionally illustrated is the similarity between the serotype M2 and GBS pilus encoding region proteins in global alignments. (B) Relationships among the FCT region variants. Nucleotide sequences bounded by the flanking conserved genes for the each of the sequenced GAS strains and the five GBS genes in panel A, were aligned with ClustalW and a neighbor-network was generated using SplitsTree.

The FCT gene region is an ∼11–16 kb region that encodes global-regulators and extracellular matrix-binding proteins involved in cell adhesion and invasion [92]–[98]. Recently, Mora et al. [99] reported that the FCT region genes encode extended pilus-like structures composed in part of polymerized T-antigen protein subunits. Importantly, immunization of laboratory animals with either GAS or GBS pilin proteins has been shown to provide protection against experimental invasive infections caused by these pathogens [99]–[101]. More recently these pilus components were shown to mediate adhesion to human pharyngeal and skin cells and participate in biofilm formation [102], [103]. We compared the FCT gene regions in the 12 sequenced GAS strains and identified six distinct variants, including four (I, II, III, and IV) that have been previously described (Fig. 6). Two variants, V in serotype M4 strain MGAS10750 and VI in serotype M2 strain MGAS10270, have not been described previously, thereby expanding our understanding of sequence variation in this region. A portion of the FCT region gene content in the serotype M2 strain is more related to genomic islands present in six sequenced GBS strains than to the other GAS variants [104], [105], consistent with the idea that horizontal gene transfer has contributed to diversification in this chromosomal segment. Thus, the serotype M2 clonal lineage has two gene regions (FCT and 10270-RD.2) that are closely related to genetic elements in GBS. The similarity of these elements between these two pathogens provides additional support for the hypothesis that the extracellular products encoded by these two regions contribute to the ability of M2 GAS strains to cause puerperal sepsis infections.

## Discussion

The 12 GAS genomes now available represent serotypes responsible for ∼70% of M protein serotypes that most commonly cause GAS pharyngitis and invasive infections in several countries in the western hemisphere [17], [18], [21], [22], [24], [45], [46], [106]. Although these 12 genome sequences provide extensive information to assist studies of virulence, development of therapeutics and diagnostics, and other aspects of GAS biology, a cautionary note is required. There is considerable variation in prophage content and prophage-associated virulence factor profile among strains of the same M type [26], [27], [29], [45], [46], [59], [106]–[109]. In addition, strains of certain M types are not necessarily clonally related [13], [56], [110]. This intra-M type genetic heterogeneity can mediate significant differences in host-pathogen interactions, as documented recently for distinct clones of serotype M1 and M3 GAS [56], [107]. Similarly, many of the genomes contain large segments of exogenous (foreign), non-prophage DNA acquired by lateral gene transfer events. In the case of serotype M1 GAS, an apparent episode of generalized transduction contributed to the evolution of a new, unusually virulent clone that increased dramatically in frequency since the mid 1980s [56]. Other foreign DNA segments may have been acquired by conjugative transposition as exemplified by the ICE-like elements we have described. Regardless of the exact gene transfer mechanism involved, the key point is that the intraspecies gene content and allelic diversity present in the GAS metagenome is extensive, and can impart important differences in disease character and epidemic behavior. The sequencing of additional GAS strains continues to reveal an unappreciated magnitude of species-level population genomic diversity.

Given the increasing prevalence of drug-resistant strains of GAS, it is important to note that these genome sequences have provided new information about the putatively-mobile genetic elements involved. The ICEs associated with genes conferring resistance to macrolide and tetracycline antibiotics are chimeric structures composed of the multiple drug-resistance genes, genetic machinery to mediate lateral transfer, and genes encoding putative or proven novel extracellular proteins [26], [83], [85]. In the case of the *mef*(A) element in serotype M6 strain MGAS10394 and other strains, it is known from serologic studies that the extracellular protein designated R6 is expressed during human infections [26], [85]. The putative extracellular proteins encoded by the *erm*(A) and *tet*(O) encoding elements, 10750-RD.2 and 2096-RD.2 respectively, have not yet been analyzed in detail. However, since they contain conventional gram-positive secretion sequences and some have carboxyterminal cell wall attachment motifs, we speculate that these proteins are either displayed on the GAS cell surface and function to mediate adherence to host molecules, or are secreted free into the extracellular environment and interact with host molecules. Given that the drug-resistance genes are widespread in Gram negative and positive respiratory tract organisms, and dispersed among many distinct GAS M protein types, we think it likely that further study will identify additional genetic elements associated with drug resistance [33]–[37], [40], [41], [43], [44], [51], [68], [69], [80], [85]–[87], [111]–[117]. Consistent with this hypothesis, an element containing both *mef*(A) and *tet*(O) genes was described recently [86]. We note that the various genetic elements associated with *tet*(O), *mef*(A), *erm*(A) likely helps to explain some of the confusing data in the literature regarding the nature and mode of spread of drug-resistant markers in GAS [68], [80]–[82], [84].

One feature of the horizontally transferred regions encoding antibiotic resistance determinants in the GAS metagenome that is of concern is the high level of homology between the genes putatively involved in mobilization and transfer of these elements and genes found in other genera and species of human pathogens such as staphylococci, enterococci, clostridia, and streptococci (*S. pneumoniae* and *S. agalactiae*, for example). Additionally the finding that the ICEs present in the GAS genome (more so than the prophages) differ significantly in nucleotide composition from the core chromosome argues that they originate from organisms not closely related to the streptococci. This underscores the potential extensiveness of accessible virulence genes, and the relative lack of barriers to horizontal gene transfer among pathogenic bacteria. The horizontally acquired chimeric elements can provide an immediate selective advantage to recipient bacteria, for example by conferring antibiotic resistance. In addition, the conserved regions of these mobile elements enhance the potential for further future recombination/integration events with horizontally transferred DNA. Thus, these foreign elements likely increase the frequency with which regions of horizontally transferred DNA are retained in the chromosome of recipient bacteria.

The addition of these four complete genome sequences to the eight previously determined makes the GAS metagenome one of the better characterized among important human pathogens. The new gene content found in the ICE-like elements and the FCT regions described in these four genomes encodes proteins providing antimicrobial resistance and proven and putative extracellular adhesin/invasin proteins. Thus the sequencing of these four additional genomes has provided much-needed information about the genetic diversity present in GAS and has revealed factors likely affecting the virulence of these strains. Although medical intervention limits human morbidity and mortality due to GAS in the western countries, globally it has a tremendous toll on human health. This is especially the case in countries with less developed medical systems for which there is relatively little information available about the circulating strains. One future challenge will be to determine to what extent the metagenome of GAS as defined by the twelve currently sequenced strains originating in the western hemisphere is representative of disease causing strains circulating in other areas of the world. Given the considerable role played by mobile exogenous elements in GAS genetic diversity and pathogenesis, this information is crucial to understanding the array of molecular mechanisms used by GAS to cause human disease, and is of paramount importance to vaccine and therapeutics research.

## Materials and Methods

### Bacterial strains

The four GAS strains sequenced, serotype M2 Strain MGAS10270, M4 strain MGAS10750, and M12 strains MGAS2096 and MGAS9429 were each isolated from human infections of defined disease type. These four strains have been deposited in the American Type Culture Collection under the following accession numbers: MGAS10270 (BAA-1063), MGAS10750 (BAA-1066), MGAS2096 (BAA-1065), and MGAS9429 (BAA-1315).

### Genome sequencing

Standard methods were used to determine the complete genome sequence of the M2, M4, and two M12 strains as previously described [26], [29]. Briefly, short sequencing templates were generated from sheared chromosomal DNA fragments cloned into a plasmid vector and sequenced from each end. The resulting random sequence reads were assembled in silico into larger contiguous segments, and contigs were ordered using the GAS metagenome as a scaffold. Sequence gaps were closed by directed sequencing of gap-spanning templates obtained by PCR amplification. Additional directed sequencing was performed as necessary to improve sequence quality genome-wide to a minimum base call error rate of 1 in 10,000 (i.e. Q40). Each genome was tiled by PCR after closure to validate the in silico assembly. Coding sequences were identified with proprietary software (Integrated Genomics, Chicago, IL), annotated, and analyzed with the ERGO bioinformatics suite [118]. The genome sequences have been deposited in the National Center for Biotechnology Information microbial genome database under the following accession numbers: MGAS10270 (CP000260), MGAS10750 (CP000262), MGAS2096 (CP000261), and MGAS9429 (CP000259). An analysis of polymorphisms present in the core chromosomes of these strains and their relationship with the other 8 sequenced GAS genomes has recently been published [119].

### Identification of endogenous and exogenous sequences

Sequence common to the GAS genomes constituting the endogenous metagenome core, were identified by a combination of genomic alignment and gene content comparisons. Exogenous putatively-mobile elements such as prophages and integrating conjugative elements (ICEs) were identified by a combination of genomic alignment, gene content, nucleotide composition, and codon usage comparisons. First, by comparative genome sequence alignments (such as MUMmer plots) these regions differ in gene content and/or context relative to one or more of the other GAS genomes. That is, they are an insertion or deletion. Second, they contain modules of genes with similarity to genes previously identified in and considered to be characteristic of mobile genetic elements. For example, prophages have genes encoding coat and tail proteins, and ICEs have genes encoding recombinases, relaxases and excisionases. In addition, prophages and ICEs were differentiated in part by the gene content they lacked, consistent with their known modes of lateral transfer. For example, unlike prophages, ICEs lack genes encoding holins and peptidoglycan lytic enzymes. Third, many ICEs and prophages are flanked by directly repeated attachment sequences, attP-L and attP-R, that are generated as a consequence of the homologous-recombination event mediated by the related prophage and ICE site-specific integrases. Fourth, these elements often contain genes that are most similar in sequence to genes of other bacterial species that sometimes differ from GAS in preferred codon usage, % G+C content, and multimer nucleotide (di-, tri-, tetra-) composition, consistent with intraspecies lateral transfer. Importantly, there is no single distinct gene complement that differentiates among various types of bacterial mobile genetic elements (phage, conjugative plasmids and transposons, insertion sequences, etc.). Thus, ICEs and prophages were identified on the basis of a preponderance of genetic characteristics identified during annotation, rather than use of a single genetic characteristic. For simplicity, integrated foreign elements that have many but not all of the features described above (that is, ICE-like or prophage-like traits) will be referred to in this report as ICEs and prophages. Together, these elements are referred to as exogenous genetic elements.

### Sequence alignments and comparisons

Genome alignments and identification of SNPs were performed using MUMmer [120]. Genomic gene content and NCBI non-redundant sequence database comparisons were performed using BLAST [121]. Pair-wise global and local gene and protein alignments were performed using the “needle” (Needleman-Wunsch) and “water” (Smith-Waterman) applications respectively of EMBOSS [122]. Multiple sequence alignments were performed using ClustalW or Muscle [123], [124]. Reconstruction of genetic relationships were performed using SplitsTree [125]. Protein motif searches were performed using the EMBL InterPro scan and NCBI conserved domain servers [126], [127]. Codon usage and nucleotide composition analyses were performed using CodonW (bioweb.pasteur.fr). Various other analyses (M.W., pI, hydrophilicity, etc.) were performed using MacVector [128]. Circular genome atlases were generated using GenomeViz [129]. Schematics of the aligned genomes were generated using the Artemis Comparison Tool [130].

## Supporting Information

Table S1 New GAS Metagenome Genes
(0.37 MB DOC)
