# Supplementary material for: Contribution of Exogenous Genetic Elements to the Group A Streptococcus Metagenome
Source: PLoS One. 2007 Aug 29;2(8):e800. doi: 10.1371/journal.pone.0000800 (PMC1949102; doi:10.1371/journal.pone.0000800)
Supplement: Table S1 — New GAS Metagenome Genes (0.37 MB DOC) [file pone.0000800.s001.doc]

**Table S1.** New GAS Metagenome Genes*

| **M-type** | **Strain** | **Locus Tag** | **Region/Element** | **Annotation** |
| --- | --- | --- | --- | --- |
| M2 | MGAS10270 | Spy_0109 | FCT region | pilus protein |
| M2 | MGAS10270 | Spy_0110 | FCT region | pilus protein |
| M2 | MGAS10270 | Spy_0111 | FCT region | sortase |
| M2 | MGAS10270 | Spy_0112 | FCT region | sortase |
| M2 | MGAS10270 | Spy_0113 | FCT region | pilus protein |
| M2 | MGAS10270 | Spy_0114 | FCT region | pilus protein |
| M2 | MGAS10270 | Spy_0115 | FCT region | sortase |
| M2 | MGAS10270 | Spy_0116 | FCT region | sortase |
| M2 | MGAS10270 | Spy_0117 | FCT region | PrtF3?, fibronectin-binding protein |
| M2 | MGAS10270 | Spy_0145 | other | hypothetical protein |
| M2 | MGAS10270 | Spy_0351 | other | hypothetical membrane associated protein |
| M2 | MGAS10270 | Spy_0355 | other | hypothetical protein |
| M2 | MGAS10270 | Spy_0356 | other | hypothetical protein |
| M2 | MGAS10270 | Spy_0360 | other | hypothetical protein |
| M2 | MGAS10270 | Spy_0490 | other | phosphoglycerate mutase family protein |
| M2 | MGAS10270 | Spy_0491 | other | hypothetical membrane spanning protein |
| M2 | MGAS10270 | Spy_0492 | other | transcriptional regulator |
| M2 | MGAS10270 | Spy_0537 | phage 10270.1 | phage protein |
| M2 | MGAS10270 | Spy_0543 | phage 10270.1 | hypothetical protein |
| M2 | MGAS10270 | Spy_0544 | phage 10270.1 | phage protein |
| M2 | MGAS10270 | Spy_0545 | phage 10270.1 | hypothetical protein |
| M2 | MGAS10270 | Spy_0893 | other | SclB, *Streptococcal* collagen-like surface protein B |
| M2 | MGAS10270 | Spy_1163 | other | Mag protein precursor |
| M2 | MGAS10270 | Spy_1164 | other | beta-N-acetylhexosaminidase |
| M2 | MGAS10270 | Spy_1165 | other | beta-N-acetylhexosaminidase |
| M2 | MGAS10270 | Spy_1166 | other | beta-N-acetylhexosaminidase |
| M2 | MGAS10270 | Spy_1167 | other | beta-N-acetylhexosaminidase |
| M2 | MGAS10270 | Spy_1168 | other | phosphoglycolate phosphatase |
| M2 | MGAS10270 | Spy_1169 | other | fructuronate reductase |
| M2 | MGAS10270 | Spy_1170 | other | mannonate dehydratase |
| M2 | MGAS10270 | Spy_1171 | other | uronate isomerase |
| M2 | MGAS10270 | Spy_1172 | other | 4-hydroxy-2-oxoglutarate aldolase |
| M2 | MGAS10270 | Spy_1173 | other | GntR family transcriptional regulator |
| M2 | MGAS10270 | Spy_1174 | other | beta-glucuronidase |
| M2 | MGAS10270 | Spy_1175 | other | 2-dehydro-3-deoxygluconokinase |
| M2 | MGAS10270 | Spy_1176 | other | sugar/sodium symporter |
| M2 | MGAS10270 | Spy_1747 | other | collagen-like surface protein |
| M2 | MGAS10270 | Spy_1756 | other | collagen-like surface protein |
| M2 | MGAS10270 | Spy_1763 | other | RhuM fragment |
| M2 | MGAS10270 | Spy_1764 | other | hypothetical protein |
| M2 | MGAS10270 | Spy_1770 | other | hypothetical protein |
| M2 | MGAS10270 | Spy_1771 | other | hypothetical membrane associated protein |
| M2 | MGAS10270 | Spy_1781 | other | YSIRK signal sequence cell surface protein |
| M2 | MGAS10270 | Spy_1783 | other | Emm2, M protein |
| M2 | MGAS10270 | Spy_1797 | other | SfbX, *Streptococcal* fibronectin-binding protein X |
| M2 | MGAS10270 | Spy_1798 | other | Sof, serum opacity factor |
| M2 | MGAS10270 | Spy_1897 | other | hypothetical protein |
| M2 | MGAS10270 | Spy_1912 | other | hypothetical protein |
| M2 | MGAS10270 | Spy_1913 | other | hypothetical protein |
| M2 | MGAS10270 | Spy_1916 | other | replication protein |
| M2 | MGAS10270 | Spy_1917 | phage 10270.5 | phage protein |
| M2 | MGAS10270 | Spy_1918 | phage 10270.5 | DNA integration/recombination/inversion protein |
| M4 | MGAS10750 | Spy_0033 | phage remnant? | putative phage resistance endonuclease |
| M4 | MGAS10750 | Spy_0034 | phage remnant? | phage-related protein |
| M4 | MGAS10750 | Spy_0114 | FCT region | PrtF1, fibronectin-binding protein |
| M4 | MGAS10750 | Spy_0115 | FCT region | PrtF3?, Fibronectin-binding protein |
| M4 | MGAS10750 | Spy_0116 | FCT region | pilus protein |
| M4 | MGAS10750 | Spy_0117 | FCT region | pilus protein |
| M4 | MGAS10750 | Spy_0118 | FCT region | sortase |
| M4 | MGAS10750 | Spy_0119 | FCT region | sortase |
| M4 | MGAS10750 | Spy_0120 | FCT region | sortase |
| M4 | MGAS10750 | Spy_0148 | other | hypothetical protein |
| M4 | MGAS10750 | Spy_0149 | other | hypothetical protein |
| M4 | MGAS10750 | Spy_0151 | other | hypothetical protein |
| M4 | MGAS10750 | Spy_0177 | other | hypothetical protein |
| M4 | MGAS10750 | Spy_0259 | other | isochorismatase |
| M4 | MGAS10750 | Spy_0354 | other | hypothetical protein |
| M4 | MGAS10750 | Spy_0355 | other | hypothetical membrane associated protein |
| M4 | MGAS10750 | Spy_0357 | other | hypothetical protein |
| M4 | MGAS10750 | Spy_0358 | other | hypothetical protein |
| M4 | MGAS10750 | Spy_0359 | other | hypothetical protein |
| M4 | MGAS10750 | Spy_0442 | other | DrrA, daunorubicin resistance ATP-binding protein |
| M4 | MGAS10750 | Spy_0443 | other | daunorubicin resistance transmembrane protein |
| M4 | MGAS10750 | Spy_0561 | phage 10750.1 | phage protein |
| M4 | MGAS10750 | Spy_0567 | phage 10750.1 | hypothetical phage protein |
| M4 | MGAS10750 | Spy_0568 | phage 10750.1 | phage protein |
| M4 | MGAS10750 | Spy_0569 | phage 10750.1 | hypothetical phage protein |
| M4 | MGAS10750 | Spy_0571 | phage 10750.1 | phage protein |
| M4 | MGAS10750 | Spy_0572 | phage 10750.1 | hypothetical phage protein |
| M4 | MGAS10750 | Spy_0573 | phage 10750.1 | phage protein |
| M4 | MGAS10750 | Spy_0657 | other | transposase |
| M4 | MGAS10750 | Spy_0658 | other | transposase |
| M4 | MGAS10750 | Spy_0928 | other | SclB, *Streptococcal* collagen-like surface protein B |
| M4 | MGAS10750 | Spy_1044 | other | hypothetical membrane associated protein |
| M4 | MGAS10750 | Spy_1679 | ICE 10750-RD.2 | replication initiation protein |
| M4 | MGAS10750 | Spy_1680 | ICE 10750-RD.2 | DnaC-like DNA replication protein |
| M4 | MGAS10750 | Spy_1681 | ICE 10750-RD.2 | hypothetical protein |
| M4 | MGAS10750 | Spy_1682 | ICE 10750-RD.2 | phage antirepressor-like protein |
| M4 | MGAS10750 | Spy_1683 | ICE 10750-RD.2 | TraG/TraD family protein |
| M4 | MGAS10750 | Spy_1684 | ICE 10750-RD.2 | hypothetical protein |
| M4 | MGAS10750 | Spy_1685 | ICE 10750-RD.2 | hypothetical protein |
| M4 | MGAS10750 | Spy_1686 | ICE 10750-RD.2 | hypothetical protein |
| M4 | MGAS10750 | Spy_1687 | ICE 10750-RD.2 | hypothetical protein |
| M4 | MGAS10750 | Spy_1688 | ICE 10750-RD.2 | pyruvate:ferrodoxin oxidoreductase beta subunit-like |
| M4 | MGAS10750 | Spy_1689 | ICE 10750-RD.2 | phosphotransferase system IIC components |
| M4 | MGAS10750 | Spy_1690 | ICE 10750-RD.2 | type IV secretory pathway, VirB4 components |
| M4 | MGAS10750 | Spy_1691 | ICE 10750-RD.2 | type IV secretory pathway, VirB4 components |
| M4 | MGAS10750 | Spy_1692 | ICE 10750-RD.2 | MutS family mismatch repair ATPase |
| M4 | MGAS10750 | Spy_1693 | ICE 10750-RD.2 | hypothetical protein |
| M4 | MGAS10750 | Spy_1694 | ICE 10750-RD.2 | putative RBC-binding protein |
| M4 | MGAS10750 | Spy_1695 | ICE 10750-RD.2 | bacteriocin-like protein |
| M4 | MGAS10750 | Spy_1696 | ICE 10750-RD.2 | DNA topoisomerase III-like protein |
| M4 | MGAS10750 | Spy_1697 | ICE 10750-RD.2 | type II restriction-modification system methylation subunit |
| M4 | MGAS10750 | Spy_1698 | ICE 10750-RD.2 | superfamily II DNA and RNA helicase, DNA methylase |
| M4 | MGAS10750 | Spy_1699 | ICE 10750-RD.2 | hypothetical protein |
| M4 | MGAS10750 | Spy_1700 | ICE 10750-RD.2 | TetR family transcriptional regulator |
| M4 | MGAS10750 | Spy_1701 | ICE 10750-RD.2 | Tetronasin resistance ATP-binding protein |
| M4 | MGAS10750 | Spy_1702 | ICE 10750-RD.2 | Tetronasin resistance transmembrane protein |
| M4 | MGAS10750 | Spy_1703 | ICE 10750-RD.2 | hypothetical protein |
| M4 | MGAS10750 | Spy_1704 | ICE 10750-RD.2 | ErmA , erythromycin resistance methyltransferase protein |
| M4 | MGAS10750 | Spy_1705 | ICE 10750-RD.2 | protein kinase-like, spectinomycin phosphotransferase? |
| M4 | MGAS10750 | Spy_1706 | ICE 10750-RD.2 | cytidine deaminase |
| M4 | MGAS10750 | Spy_1707 | ICE 10750-RD.2 | Zn-dependent hydrolase, metallo-beta-lactamase family |
| M4 | MGAS10750 | Spy_1708 | ICE 10750-RD.2 | relaxase/mobilization nuclease domain |
| M4 | MGAS10750 | Spy_1709 | ICE 10750-RD.2 | putative maturase/reverse transcriptase |
| M4 | MGAS10750 | Spy_1710 | ICE 10750-RD.2 | relaxase/mobilization nuclease domain |
| M4 | MGAS10750 | Spy_1711 | ICE 10750-RD.2 | hypothetical protein |
| M4 | MGAS10750 | Spy_1712 | ICE 10750-RD.2 | hypothetical protein |
| M4 | MGAS10750 | Spy_1713 | ICE 10750-RD.2 | hypothetical protein |
| M4 | MGAS10750 | Spy_1714 | ICE 10750-RD.2 | putative HTH DNA-binding transcriptional regulator |
| M4 | MGAS10750 | Spy_1715 | ICE 10750-RD.2 | hypothetical protein |
| M4 | MGAS10750 | Spy_1716 | ICE 10750-RD.2 | hypothetical protein |
| M4 | MGAS10750 | Spy_1717 | ICE 10750-RD.2 | site-specific recombinase |
| M4 | MGAS10750 | Spy_1718 | ICE 10750-RD.2 | site-specific recombinase |
| M4 | MGAS10750 | Spy_1719 | ICE 10750-RD.2 | site-specific recombinase |
| M4 | MGAS10750 | Spy_1781 | other | SclA, *Streptococcal* collagen-like surface protein A |
| M4 | MGAS10750 | Spy_1806 | other | YSIRK signal sequence cell surface protein |
| M4 | MGAS10750 | Spy_1809 | other | Arp4, M protein like IgA receptor |
| M4 | MGAS10750 | Spy_1822 | other | SfbX, *Streptococcal* fibronectin-binding protein X |
| M4 | MGAS10750 | Spy_1823 | other | Sof, serum opacity factor |
| M4 | MGAS10750 | Spy_1899 | phage 10750.4 | Cro/CI family transcriptional regulator |
| M4 | MGAS10750 | Spy_1900 | phage 10750.4 | Cro/CI family transcriptional regulator |
| M4 | MGAS10750 | Spy_1910 | phage 10750.4 | DNA primase |
| M4 | MGAS10750 | Spy_1913 | phage 10750.4 | phage protein |
| M4 | MGAS10750 | Spy_1914 | phage 10750.4 | phage protein |
| M4 | MGAS10750 | Spy_1915 | phage 10750.4 | hypothetical protein |
| M4 | MGAS10750 | Spy_1916 | phage 10750.4 | hypothetical protein |
| M4 | MGAS10750 | Spy_1917 | phage 10750.4 | phage protein |
| M4 | MGAS10750 | Spy_1918 | phage 10750.4 | phage protein |
| M4 | MGAS10750 | Spy_1919 | phage 10750.4 | hypothetical protein |
| M12 | MGAS2096 | Spy_0110 | FCT region | fibronectin-binding protein |
| M12 | MGAS2096 | Spy_0113 | FCT region | fibronectin-binding protein |
| M12 | MGAS2096 | Spy_0115 | FCT region | fibronectin-binding protein |
| M12 | MGAS2096 | Spy_0155 | other | transposase |
| M12 | MGAS2096 | Spy_0371 | other | hypothetical membrane associated protein |
| M12 | MGAS2096 | Spy_0372 | other | hypothetical membrane associated protein |
| M12 | MGAS2096 | Spy_0373 | other | hypothetical membrane associated protein |
| M12 | MGAS2096 | Spy_0376 | other | hypothetical protein |
| M12 | MGAS2096 | Spy_0757 | other | transposase |
| M12 | MGAS2096 | Spy_0851 | other | SclB, *Streptococcal* collagen-like surface protein B |
| M12 | MGAS2096 | Spy_1103 | ICE 2096-RD.2 | site-specific recombinase |
| M12 | MGAS2096 | Spy_1104 | ICE 2096-RD.2 | MazF protein |
| M12 | MGAS2096 | Spy_1105 | ICE 2096-RD.2 | RNA polymerase ECF-type sigma factor |
| M12 | MGAS2096 | Spy_1106 | ICE 2096-RD.2 | hypothetical protein |
| M12 | MGAS2096 | Spy_1107 | ICE 2096-RD.2 | two component system histidine kinase |
| M12 | MGAS2096 | Spy_1108 | ICE 2096-RD.2 | two-component response regulator |
| M12 | MGAS2096 | Spy_1109 | ICE 2096-RD.2 | two-component response regulator |
| M12 | MGAS2096 | Spy_1110 | ICE 2096-RD.2 | ABC transporter permease protein |
| M12 | MGAS2096 | Spy_1111 | ICE 2096-RD.2 | ABC transporter ATP-binding protein |
| M12 | MGAS2096 | Spy_1112 | ICE 2096-RD.2 | transcriptional regulator |
| M12 | MGAS2096 | Spy_1113 | ICE 2096-RD.2 | Na+ driven multidrug efflux pump |
| M12 | MGAS2096 | Spy_1114 | ICE 2096-RD.2 | hypothetical protein |
| M12 | MGAS2096 | Spy_1115 | ICE 2096-RD.2 | ferredoxin |
| M12 | MGAS2096 | Spy_1116 | ICE 2096-RD.2 | TetR family transcriptional regulator |
| M12 | MGAS2096 | Spy_1117 | ICE 2096-RD.2 | hypothetical protein |
| M12 | MGAS2096 | Spy_1118 | ICE 2096-RD.22 | virginiamycin A acetyltransferase |
| M12 | MGAS2096 | Spy_1119 | ICE 2096-RD.2 | hypothetical protein |
| M12 | MGAS2096 | Spy_1120 | ICE 2096-RD.2 | MerR family transcriptional regulator |
| M12 | MGAS2096 | Spy_1121 | ICE 2096-RD.2 | antigen |
| M12 | MGAS2096 | Spy_1122 | ICE 2096-RD.2 | sortase B family protein |
| M12 | MGAS2096 | Spy_1123 | ICE 2096-RD.2 | hypothetical protein |
| M12 | MGAS2096 | Spy_1124 | ICE 2096-RD.2 | adenine-specific methyltransferase |
| M12 | MGAS2096 | Spy_1125 | ICE 2096-RD.2 | TRSE protein |
| M12 | MGAS2096 | Spy_1126 | ICE 2096-RD.2 | hypothetical protein |
| M12 | MGAS2096 | Spy_1127 | ICE 2096-RD.2 | hypothetical protein |
| M12 | MGAS2096 | Spy_1128 | ICE 2096-RD.2 | adenine-specific methyltransferase |
| M12 | MGAS2096 | Spy_1129 | ICE 2096-RD.2 | aspartyl/glutamyl-tRNA amidotransferase subunit A |
| M12 | MGAS2096 | Spy_1130 | ICE 2096-RD.2 | TraG/TraD family protein |
| M12 | MGAS2096 | Spy_1131 | ICE 2096-RD.2 | hypothetical protein |
| M12 | MGAS2096 | Spy_1132 | ICE 2096-RD.2 | hypothetical protein |
| M12 | MGAS2096 | Spy_1133 | ICE 2096-RD.2 | relaxase |
| M12 | MGAS2096 | Spy_1134 | ICE 2096-RD.2 | relaxosome component |
| M12 | MGAS2096 | Spy_1135 | ICE 2096-RD.2 | LtrC-like protein |
| M12 | MGAS2096 | Spy_1136 | ICE 2096-RD.2 | hypothetical protein |
| M12 | MGAS2096 | Spy_1137 | ICE 2096-RD.2 | hypothetical protein |
| M12 | MGAS2096 | Spy_1138 | ICE 2096-RD.2 | hypothetical protein |
| M12 | MGAS2096 | Spy_1139 | ICE 2096-RD.2 | superfamily II DNA and RNA helicase (SNF2 family) |
| M12 | MGAS2096 | Spy_1140 | ICE 2096-RD.2 | site-specific recombinase |
| M12 | MGAS2096 | Spy_1141 | ICE 2096-RD.2 | hypothetical cytosolic protein |
| M12 | MGAS2096 | Spy_1142 | ICE 2096-RD.2 | ParB, chromosome partitioning protein |
| M12 | MGAS2096 | Spy_1143 | ICE 2096-RD.2 | Mob family plasmid recombination protein |
| M12 | MGAS2096 | Spy_1144 | ICE 2096-RD.2 | hypothetical protein |
| M12 | MGAS2096 | Spy_1145 | ICE 2096-RD.2 | RNA polymerase ECF-type sigma factor |
| M12 | MGAS2096 | Spy_1146 | ICE 2096-RD.2 | hypothetical cytosolic protein |
| M12 | MGAS2096 | Spy_1147 | ICE 2096-RD.2 | RNA polymerase sigma-B factor |
| M12 | MGAS2096 | Spy_1148 | ICE 2096-RD.2 | hypothetical protein |
| M12 | MGAS2096 | Spy_1149 | ICE 2096-RD.2 | TetO, tetracycline resistance protein |
| M12 | MGAS2096 | Spy_1150 | ICE 2096-RD.2 | TnpV-like transposase |
| M12 | MGAS2096 | Spy_1151 | ICE 2096-RD.2 | superfamily II DNA and RNA helicase (SNF2 family) |
| M12 | MGAS2096 | Spy_1152 | ICE 2096-RD.2 | hypothetical protein |
| M12 | MGAS2096 | Spy_1153 | ICE 2096-RD.2 | hypothetical protein |
| M12 | MGAS2096 | Spy_1154 | ICE 2096-RD.2 | hypothetical protein |
| M12 | MGAS2096 | Spy_1155 | ICE 2096-RD.2 | hypothetical membrane associated protein |
| M12 | MGAS2096 | Spy_1156 | ICE 2096-RD.2 | collagen adhesion protein |
| M12 | MGAS2096 | Spy_1157 | ICE 2096-RD.2 | ParB, chromosome partitioning protein B |
| M12 | MGAS2096 | Spy_1158 | ICE 2096-RD.2 | ParA, chromosome partitioning protein A |
| M12 | MGAS2096 | Spy_1159 | ICE 2096-RD.2 | hypothetical protein |
| M12 | MGAS2096 | Spy_1565 | other | endo-beta-N-acetylglucosaminidase F2 precursor |
| M12 | MGAS2096 | Spy_1718 | other | RhuM fragment |
| M12 | MGAS2096 | Spy_1719 | other | ArpU family transcriptional regulator |
| M12 | MGAS2096 | Spy_1720 | other | transposase |
| M12 | MGAS2096 | Spy_1721 | other | hypothetical protein |
| M12 | MGAS2096 | Spy_1722 | other | hypothetical protein |
| M12 | MGAS2096 | Spy_1723 | other | hypothetical protein |
| M12 | MGAS2096 | Spy_1747 | other | Sic, *Streptococcal* inhibitor of complement |
| M12 | MGAS2096 | Spy_1762 | other | Sof, serum opacity factor |
| M12 | MGAS9429 | Spy_0108 | FCT region | PrtF1, fibronectin-binding protein |
| M12 | MGAS9429 | Spy_0111 | FCT region | Cpa |
| M12 | MGAS9429 | Spy_0113 | FCT region | pilus protein |
| M12 | MGAS9429 | Spy_0355 | other | hypothetical membrane associated protein |
| M12 | MGAS9429 | Spy_0356 | other | hypothetical membrane associated protein |
| M12 | MGAS9429 | Spy_0360 | other | hypothetical protein |
| M12 | MGAS9429 | Spy_0534 | phage 9429.1 | phage protein |
| M12 | MGAS9429 | Spy_0540 | phage 9429.1 | hypothetical phage protein |
| M12 | MGAS9429 | Spy_0541 | phage 9429.1 | phage protein |
| M12 | MGAS9429 | Spy_0542 | phage 9429.1 | hypothetical phage protein |
| M12 | MGAS9429 | Spy_0544 | phage 9429.1 | phage protein |
| M12 | MGAS9429 | Spy_0545 | phage 9429.1 | hypothetical phage protein |
| M12 | MGAS9429 | Spy_0546 | phage 9429.1 | phage protein |
| M12 | MGAS9429 | Spy_0741 | other | transposase |
| M12 | MGAS9429 | Spy_0796 | phage 9429.2 | hypothetical phage protein |
| M12 | MGAS9429 | Spy_0797 | phage 9429.2 | phage transcriptional repressor |
| M12 | MGAS9429 | Spy_0844 | phage 9429.2 | phage infection protein |
| M12 | MGAS9429 | Spy_0894 | other | SclB, *Streptococcal* collagen-like surface protein B |
| M12 | MGAS9429 | Spy_1543 | other | endo-beta-N-acetylglucosaminidase F2 precursor |
| M12 | MGAS9429 | Spy_1696 | other | RhuM fragment |
| M12 | MGAS9429 | Spy_1697 | other | ArpU family phage encoded transcriptional regulator |
| M12 | MGAS9429 | Spy_1698 | other | transposase |
| M12 | MGAS9429 | Spy_1699 | other | hypothetical protein |
| M12 | MGAS9429 | Spy_1700 | other | hypothetical protein |
| M12 | MGAS9429 | Spy_1722 | other | Sic, *Streptococcal* inhibitor of complement |
| M12 | MGAS9429 | Spy_1737 | other | Sof, serum opacity factor |

*Each of these genes shares less than 50% nucleotide identity throughout its full length in BLASTN comparative alignment with the eight previously determined GAS genomes.
